# Clinical Outcomes After Joint-Preserving and Joint-Sacrificing Surgery for Hallux Rigidus

**DOI:** 10.7759/cureus.42155

**Published:** 2023-07-19

**Authors:** Don Koh, Darshana Chandrakumara, Raj Socklingam, Charles Kon Kam King

**Affiliations:** 1 Orthopaedics, Changi General Hospital, Singapore, SGP; 2 Orthopaedic Surgery, Changi General Hospital, Singapore, SGP

**Keywords:** osteoarthritis (oa), foot and ankle reconstruction, first metatarsophalangeal arthrodesis, hallux disorders, ankle and foot, hallux rigidus

## Abstract

Introduction

Hallux rigidus (HR) is a degenerative condition affecting the first metatarsal phalangeal joint, causing stiffness and pain. Surgery is indicated for those who have failed a trial of conservative management. The purpose of this paper is to evaluate the functional outcomes at short and medium term after surgery for HR.

Methods

All patients who underwent surgical treatment for HR between 2017 and 2022 at the time of this study were identified and invited to return for a follow-up evaluation. Outcomes were assessed by comparison of pre-operative and post-operative visual analogue scale (VAS) and American Orthopaedic Foot and Ankle Society (AOFAS) scores.

Results

A total of 26 patients were included in our study with a mean follow-up of 31 months. There was a mean improvement in VAS score by 5.6 (p-value < 0.0001) and 5.7 (p-value = 0.0012) in patients who underwent joint-preserving (JP) and joint-sacrificing (JS) surgery, respectively. Patients who underwent JP surgery had a mean increase of 28.1 points (p-value < 0.0001) in the AOFAS Hallux score, while patients who underwent JS surgery had a mean increase of 27.29 points (p-value = 0.0066).

Conclusion

Functional outcomes after surgical management for HR are good at short- and medium-term follow-up. Good outcomes are seen with both JP and JS procedures. JP procedures should be considered as a first-line surgical option for HR as it allows revision procedures if required.

## Introduction

Hallux rigidus (HR) refers to osteoarthritis (OA) of the first metatarsal phalangeal joint (MTPJ) and is the most common arthritic condition affecting the foot [1]. The prevalence of HR in individuals older than 50 years in the Asian population was reported to be 26.7% based on radiographic imaging, with 16.4% of those being symptomatic [2]. The most common etiology of HR is idiopathic, followed by trauma to the joint. Other causes of degenerative disease in the joint include repetitive stress or inflammatory conditions such as gout and rheumatoid arthritis. Development of osteophytes and narrowing of the joint space leads to symptoms, such as reduced range of motion and debilitating pain at the first MTPJ, often resulting in altered gait mechanics, which can lead to the development of pain elsewhere [3,4].

Diagnosis of HR begins with history-taking and physical examination, along with plain film radiographs. A history of previous trauma to the joint may be elicited, and the finding of a tender first MTPJ with reduced and painful range of motion is often seen. Radiographic findings of HR include joint space narrowing and osteophyte formation between the first metatarsal head and proximal phalanx, as well as development of subchondral sclerosis and cysts [5].

As with other degenerative joint conditions, management can be either non-surgical or surgical. Conservative management includes the use of intra-articular injections, physiotherapy, shockwave therapy, and podiatry for footwear, insoles, and orthotics. Although the evidence supporting the aforementioned measures for HR is poor, patients should still be offered a trial of conservative management before considering surgery [6].

Surgical management is indicated for those who have failed non-surgical management and can be divided into joint-preserving (JP) and joint-sacrificing (JS) procedures. JP procedures, as the name suggests, conserve the anatomical joint and include cheilectomy and osteotomy. JS procedures, on the other hand, involve either replacement of the joint with arthroplasty, or fusion with arthrodesis.

Our study thus aims to evaluate the clinical outcomes following JP surgery and JS surgery for HR.

## Materials and methods

Methods

All patients who underwent surgical treatment for HR between 2017 and 2022 at the author’s hospital by an experienced foot and ankle surgeon (C.K.) were retrospectively enrolled in this study. After receiving the Institutional Review Board (IRB) approval for this study, the clinical and operative notes, and X-ray reports were retrospectively analyzed. Patients who suffered new injuries to the operated limb were excluded from the study. Patients were called back for a final assessment at the time of this review, with clinical and functional outcomes evaluated using the visual analogue scale (VAS) score and the American Orthopaedic Foot and Ankle Society (AOFAS) Hallux score.

This study was approved by the SingHealth IRB (approval number 2021/2629).

Radiographic classification

X-rays of the operated foot were retrospectively analyzed separately by the authors, and the severity of HR was graded based on the classification described by Hattrup and Johnson, as shown in Table 1 [7]. In the event of discrepancy in grading, the authors would convene and agree upon a grade.

**Table 1:** Hattrup and Johnson's radiographic classification of hallux rigidus

Grade	Radiographic findings
1	Mild to moderate osteophyte formation, preservation of joint space
2	Moderate osteophyte formation, joint space narrowing, subchondral sclerosis
3	Marked osteophyte formation, severe loss of joint space, subchondral cyst formation

Scores

Two different scoring systems were used to evaluate and compare clinical outcomes. The VAS score is a scale rating from 1 to 10 devised for patients to report the severity of their pain [8]. The AOFAS Hallux Score evaluates the functional status of the hallux and the first metatarsal by incorporating both subjective and objective information, with a score ranging from 0 to 100. Patients were evaluated using the two scores based on symptoms experienced pre-surgery and at the time of follow-up.

Statistics

Quantitative results are reported as mean (SD) with 95% confidence intervals (CI) calculated.

## Results

Patients

The study included a total of 26 patients, comprising 13 males and 13 females. Surgery was performed on a total of 27 first MTPJs, with 20 being on the right side and 7 on the left side. The average age of patients at the time of surgery was 58 years (range: 35-73 years), and the average body mass index (BMI) at the time of surgery was 26.5 kg/m2 (range: 18.7-36.6 kg/m2). The etiology of HR was idiopathic in all cases except for two first MTPJs belonging to the same patient. Furthermore, 44.4% (12/27) and 55.6% (15/27) of cases were grade 2 and grade 3 HR based on radiographic classification, respectively. The mean follow-up was 31 months (range: 8-69 months). Table 2 provides a summary of the aforementioned information.

**Table 2:** Patient demographics

Measurement		Value
No. of patients		26
No. of the first metatarsophalangeal joints		27
Age (year)		58 ± 11.4 (range: 35-73 years)
Sex	Male	13 (50.0%)
	Female	13 (50.0%)
Body mass index (kg/m^2^)		26.5 ± 4.2 (range: 18.7-36.6 kg/m^2^)
Laterality	Right	20 (74.1%)
	Left	7 (25.9%)
Etiology	Idiopathic	25 (92.6%)
	Gout	2 (7.4%)
Radiographic classification	Grade 2	12 (44.4%)
	Grade 3	15 (55.6%)
Time to follow-up (months)		31.3 ± 15.1 (range: 8-69)

Surgical procedures

For JP surgery, the procedure of choice was a cheilectomy, with associated procedures such as subchondral arthroplasty, interposition arthroplasty, and platelet-rich plasma therapy performed additionally if indicated. In terms of JS surgery, the preferred technique was arthrodesis with plating and screws. Overall, 74% (20 of 27) patients underwent JP surgery, while the remaining 26% (7 of 27) patients underwent JS arthrodesis. The aforementioned information is shown in Table 3.

**Table 3:** Surgical procedures

Number of patients	Procedure
20 (74%)	Joint preserving: cheilectomy
7 (26%)	Joint sacrificing: arthrodesis

VAS scores

**Figure 1:**
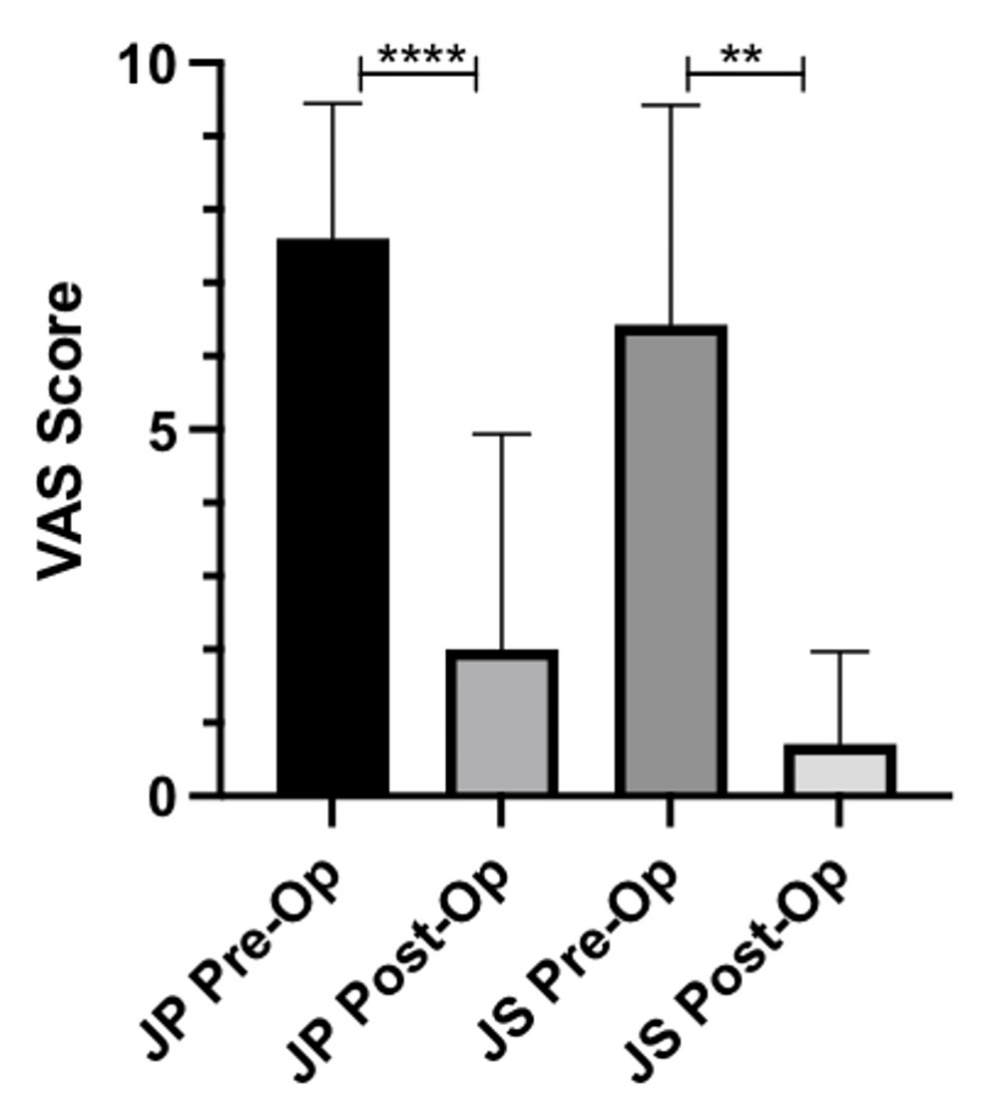
Pre- and post-operative VAS scores VAS, visual analogue scale; JP, joint preserving; JS, joint sacrificing **p-value < 0.01; ****p-value < 0.001

There was a mean improvement in VAS score by 5.6 (p-value < 0.0001) in patients who underwent JP surgery. Patients who underwent JS surgery also had a mean improvement in VAS scores by 5.7 (p-value = 0.0012). The information is displayed in Figure 1 and summarized in Table 4.

**Table 4:** Clinical outcomes AOFAS, American Orthopaedic Foot and Ankle Society; JP, joint preserving; JS, joint sacrificing; SD, standard deviation; VAS, visual analogue score

Measurement	Value	P-values
VAS score pre-operatively	JP: 7.6 ± 1.8 (SD)	-
	JS: 6.4 ± 2.8 (SD)	-
VAS score post-operatively	JP: 2.0 ± 2.9 (SD)	-
	JS: 0.7 ± 1.2 (SD)	-
Change in VAS score	JP: 5.6 (95% CI: 4.27–6.93)	<0.0001
	JS: 5.7 (95% CI: 3.28–8.14)	0.0012
AOFAS hallux score pre-operatively	JP: 60.3 ± 9.8 (SD)	-
	JS: 61.3 ± 18.7 (SD)	-
AOFAS hallux score post-operatively	JP: 88.3 ± 13.0 (SD)	-
	JS: 88.6 ± 7.4 (SD)	-
Change in AOFAS hallux score	JP: 28.1 (95% CI: 22.21–33.89)	<0.0001
	JS: 27.29 (95% CI: 10.85–43.82)	0.0066

AOFAS hallux scores

**Figure 2:**
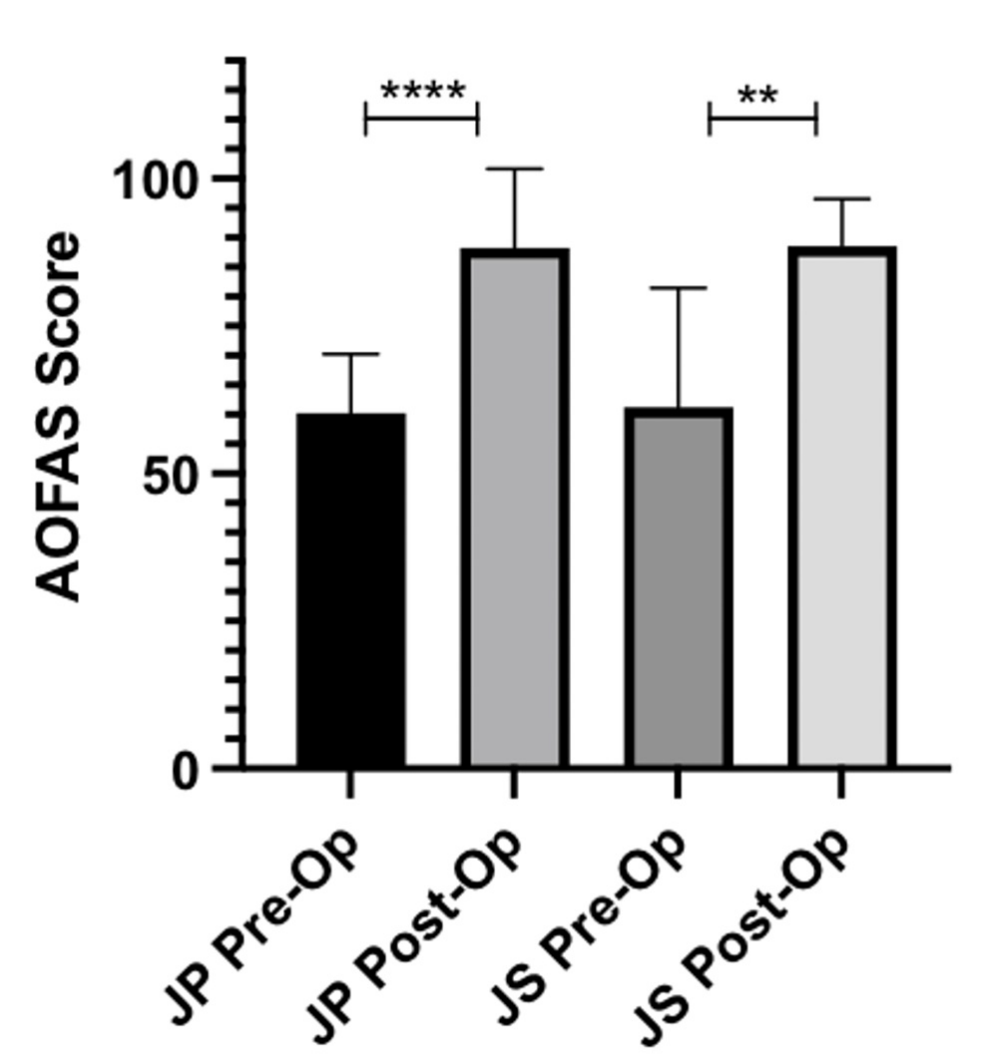
Pre- and post-operative AOFAS hallux scores AOFAS, American Orthopaedic Foot and Ankle Society; JP, joint preserving; JS, joint sacrificing **p-value < 0.01; ****p-value < 0.001

Patients who underwent JP surgery had a mean increase in AOFAS Hallux scores of 28.1 points (p-value < 0.0001). There was a mean increase in AOFAS Hallux scores of 27.29 points (p-value = 0.0066) in patients who underwent JS surgery. The aforementioned information is displayed in Figure 2 and summarized in Table 4.

## Discussion

Good outcomes were seen in our patients after both cheilectomy and arthrodesis. Improvement in both functional and pain scores after surgical management for HR is consistent with the existing literature.

Arthrodesis has been widely considered to be the treatment of choice for grade 3 HR [9,10] despite complications such as non-union being as high as 20% and functional complaints of joint stiffness and metatarsalgia [11]. The JS nature of the surgery leaves little room for revision procedures if required.

Cheilectomy is an alternative surgical option, which confers its own advantages over arthrodesis. The JP nature has shown a faster return to daily activities after surgery, along with lower complication rates [12]. Perhaps the biggest advantage of JP surgery lies in the preservation of the anatomy, allowing additional procedures to still be performed, including JS options.

Our study also suggests that cheilectomy has good outcomes even in patients with higher grade HR. Other outcome studies have also similarly reported improved range of motion, subjective functional scores, and reduction in pain for patients with higher grade of HR who undergo cheilectomy [13].

While there is general agreement that arthrodesis is the gold standard treatment for HR, consideration should be made to explore the option of cheilectomy as the first-line surgical treatment. Cheilectomy can be performed via a minimally invasive approach, conferring its own benefits compared to open surgery. Good clinical outcomes have also recently been reported when performed in conjunction with a first MTPJ arthroscopy [14]. Additionally, good outcomes have also been reported when associated procedures are performed in conjunction with the traditional cheilectomy, such as interposition arthroplasty [15], and biologic augmentation.

Furthermore, as mentioned earlier, performing cheilectomy in the initial setting would still allow room for conversion to arthrodesis later on in the event of poor outcomes. Treatment decisions, however, would still require comprehensive patient counselling about the potential need for a subsequent procedure in the event of poor outcome as long-term data regarding outcomes after JP surgery are still lacking. Further studies are also required to compare clinical outcomes between the various associated procedures to determine the gold standard adjunct for cheilectomy.

Limitations

Our study is not without limitations. Firstly, pre-operative VAS scores and AOFAS Hallux scores were retrospectively calculated at the time of final follow-up, possibly leading to variability in the patient self-reported scorings. Secondly, our study only extends toward the South East Asian population and includes a relatively small sample size. Lastly, there was also a variability in terms of the associated procedures that were performed alongside cheilectomy. Further studies should be conducted to evaluate the efficacy of each of these associated procedures.

## Conclusions

Surgical management for HR shows good outcomes at short- and medium-term follow-up in both pain and functional scoring. Good outcomes are seen after both JP and JS procedures, and either can be considered as a feasible option for surgery. JP procedures should be considered as a first-line option with regard to surgery for HR as it allows room for revision procedures in the event of poorer outcomes. Further studies, however, are still required to assess the outcomes after JS surgery in the long term to support its use over JS surgery as the first-line treatment for HR.

## References

[1] Ho B, Baumhauer J (2017). Hallux rigidus. EFORT Open Rev.

[2] Senga Y, Nishimura A, Ito N, Kitaura Y, Sudo A (2021). Prevalence of and risk factors for hallux rigidus: a cross-sectional study in Japan. BMC Musculoskelet Disord.

[3] Jacob HA (2001). Forces acting in the forefoot during normal gait--an estimate. Clin Biomech (Bristol, Avon).

[4] Zammit GV, Menz HB, Munteanu SE (2009). Structural factors associated with hallux limitus/rigidus: a systematic review of case control studies. J Orthop Sports Phys Ther.

[5] Hanft JR, Mason ET, Landsman AS, Kashuk KB (1993). A new radiographic classification for hallux limitus. J Foot Ankle Surg.

[6] Kon Kam King C, Loh Sy J, Zheng Q, Mehta KV (2017). Comprehensive review of non-operative management of hallux rigidus. Cureus.

[7] Hattrup SJ, Johnson KA (1988). Subjective results of hallux rigidus following treatment with cheilectomy. Clin Orthop Relat Res.

[8] Couper MP, Tourangeau R, Conrad FG (2006). Evaluating the effectiveness of visual analog scales: a web experiment. Soc Sci Comput Rev.

[9] Munteanu SE, Zammit GV, Menz HB, Landorf KB, Handley CJ, Elzarka A, Deluca J (2011). Effectiveness of intra-articular hyaluronan (Synvisc, hylan G-F 20) for the treatment of first metatarsophalangeal joint osteoarthritis: a randomised placebo-controlled trial. Ann Rheum Dis.

[10] Peace RA, Hamilton GA (2012). End-stage hallux rigidus: cheilectomy, implant, or arthrodesis?. Clin Podiatr Med Surg.

[11] Faraj AA, Naraen A, Twigg P (2007). A comparative study of wire fixation and screw fixation in arthrodesis for the correction of hallux rigidus using an in vitro biomechanical model. Foot Ankle Int.

[12] Bussewitz BW, Dyment MM, Hyer CF (2013). Intermediate-term results following first metatarsal cheilectomy. Foot Ankle Spec.

[13] Nicolosi N, Hehemann C, Connors J, Boike A (2015). Long-term follow-up of the cheilectomy for degenerative joint disease of the first metatarsophalangeal joint. J Foot Ankle Surg.

[14] Glenn RL, Gonzalez TA, Peterson AB, Kaplan J (2021). Minimally invasive dorsal cheilectomy and hallux metatarsal phalangeal joint arthroscopy for the treatment of hallux rigidus. Foot Ankle Orthop.

[15] Aynardi MC, Atwater L, Dein EJ, Zahoor T, Schon LC, Miller SD (2017). Outcomes after interpositional arthroplasty of the first metatarsophalangeal joint. Foot Ankle Int.

